# Electromyographic Prediction of Walking Independence in Patients With Incomplete Spinal Cord Injury

**DOI:** 10.1016/j.arrct.2025.100533

**Published:** 2025-10-13

**Authors:** Tatsuya Sugimoto, Yuma Sonoda, Nobuhito Taniguchi, Hiroshi Kawaguchi, Shintaro Izumi

**Affiliations:** a Department of Rehabilitation, Japanese Red Cross Kobe Hospital, Kobe, Japan.; b Kobe University Graduate School of Science Technology and Innovation, Kobe, Japan.; c Kobe University Graduate School of Health Sciences, Kobe, Japan.; d Osaka Heat Cool Inc, Osaka, Japan.

**Keywords:** Spinal cord injuries, Electromyography, Walking, Predictive learning models, Rehabilitation

## Abstract

**Objective:**

To predict walking independence in patients with incomplete cervical cord injury (ICCI) using electromyography of the trunk and lower extremity during straight-leg raising (SLR).

**Design:**

Prospective cohort study. Prediction model using logistic regression.

**Setting:**

Single acute-care hospital.

**Participants:**

Forty patients equally split between walking dependent and walking independent groups (mean ages: 70.7±14.4 and 63.7±15.4 years; male-to-female ratios: 18:2 and 16:4; total length of stay: 31.2±20.9 and 25.7±16.5 days).

**Interventions:**

Not applicable.

**Main Outcome Measures:**

Trunk and lower extremity acceleration and surface electromyography data measured during nondominant SLR and basic information were used to predict the level of walking independence at discharge or transfer from the acute-care hospital.

**Results:**

The results showed that the model with the lower extremity motor score (LEMS) and the root mean square (RMS) of the contralateral external oblique (EO) had the lowest Akaike information criterion of 22.24, and both were significant factors in predicting walking independence at discharge or transfer from the acute-care hospital (*P*=.019 and .034). The predictive accuracy, sensitivity, specificity, and area under the receiver operating characteristic curve were 0.875, 0.850, 0.900, and 0.975 (95% confidence interval: 0.939-1.000), respectively.

**Conclusions:**

A higher LEMS and lower RMS of the contralateral EO during nondominant SLR are significant predictors of walking independence at discharge or transfer from an acute-care hospital in patients with ICCI.

With the aging of populations worldwide, spinal cord injuries have become more common recently among elderly individuals, and the incomplete cervical cord injury (ICCI) has been increasing.^1^ The crucial challenge in the rehabilitation of these patients is eventual independent walking. Previous studies have reported that age and peripheral muscle strength are predictors of walking independence.^2^, ^3^, ^4^ However, in clinical practice, we have observed cases in which the peripheral muscle strength has improved but deviates from the level of independence in activities of daily living, leading us to suspect a decline in trunk function. Therefore, we believe that walking independence can be predicted more accurately by assessing both trunk and lower extremity strength.

We focused on straight-leg raising (SLR) exercises to evaluate both trunk and lower extremity muscles. Previous studies have reported healthy subjects activate not only the rectus femoris (RF) and iliopsoas, the primary muscles responsible for hip flexion, but also the contralateral biceps femoris (BF) and bilateral internal oblique (IO) muscles to stabilize the pelvis during SLR.^5^, ^6^, ^7^, ^8^ Additionally, to a lesser extent, the rectus abdominis (RA) and external oblique (EO) muscles are predominantly activated on the exercise-side. By contrast, patients with chronic orthopedic pain have been reported to exhibit increased activity of the RF, bilateral EO, and exercise-side IO and decreased activity of the contralateral IO compared with healthy subjects.^5^^,^^9^^,^^10^ Therefore, muscle activity alterations during SLR may be more pronounced in patients with ICCI who have generalized motor dysfunction and vary according to the walking independence.

Based on this idea, we previously performed continuous wavelet transforms on surface electromyography (EMG) during SLR and compared the temporal changes in each frequency band separately for independent and nonindependent walking groups.^11^ The results showed that the independent walking group had higher values of the high-frequency component of the BF, which is considered to reflect type II fiber activity. In contrast, the nonindependent walking group had higher values of the low-frequency component of the BF, which is considered to reflect type I fiber activity, as well as both the high- and low-frequency components of the RA and EO. These suggest that the muscle fiber types recruited during SLR may differ depending on the level of walking independence, with the independent group primarily activating the lower extremity muscles, whereas the nonindependent group engaged both the trunk and lower extremity muscles. These results were more pronounced during SLR on the nondominant legs.

However, in our previous study, we only performed group comparisons for each EMG item and were unable to perform a multivariate analysis with walking independence as the objective variable because of the small sample size. In the present study, we increased the sample size to construct a predictive model using multivariate analysis. For this purpose, we relaxed the inclusion criteria to include those patients who acquired SLR only in their nondominant legs and by continuing to measure SLR. Therefore, this study aimed to predict walking independence at discharge or transfer from an acute-care hospital for patients with ICCI using EMG of the trunk and lower extremity during SLR.

## Methods

### Patients

This study was designed as a prospective cohort study. Forty patients with ICCI hospitalized between March 2021 and November 2024 at a single acute-care hospital in Kobe, Japan, were included in this study. No a priori power calculation was done; sample size was based on available cases in this exploratory study. The inclusion criteria comprised patients diagnosed with grades C or D (motor incomplete) in the American Spinal Injury Association Impairment Scale (AIS) published in 2019 while admitted to the acute-care hospital. All patients were capable of independent walking and activities of daily living before the injury without discernible cognitive decline. The exclusion criteria included complications or a history of disease that could interfere with the measurement. All procedures were performed in accordance with the ethical standards of the institutional and national research committee and with the 1964 Helsinki Declaration and its later amendments or comparable ethical standards. This study was approved by the Medical Ethics Committee of the Japanese Red Cross Kobe Hospital (registry number: 200). All patients received both verbal and written explanations of the study, and written informed consent was obtained from each patient.

After admission, patients were evaluated for SLR acquisition on each side. The criterion was the ability to raise their legs to 30° and hold them for 5 s.^12^ We assessed the upper extremity motor score (UEMS) and lower extremity motor score (LEMS), as proposed by the American Spinal Injury Association, either before or after the SLR measurement, to evaluate the overall peripheral muscle strength. The level of walking independence upon discharge or transfer from the acute-care hospital was assessed on a scale of 0 to 8 points using item 12 (“Mobility Indoors”) of the Spinal Cord Independence Measure (SCIM).^13^ Patients were classified into 2 groups according to their scores: walking dependent (WD) and walking independent (WI) groups. The WD group required supervision or were unable to walk, with a score of 3 or less. The WI group could walk independently with or without aid, with a score of 4 or more.^2^^,^^11^^,^^14^

### Measurement procedures

After first assessing whether patients had acquired SLR on their nondominant legs, we measured data during the nondominant SLR based on our previous findings.^11^ To determine the dominant leg, patients were asked to self-report to a question on which leg they used to kick a ball. The starting position was the supine position, with the legs straight on a treatment bed. They were instructed to raise their nondominant leg to 30° at their normal speed without bending the knee and to hold it for 10 s. They performed this task in 3 trials with at least 1 min of rest between trials. They were shown the raising angle in advance and practiced it as required.

### Instruments

Three inertial measurement units with built-in triaxial accelerometers and gyroscopes (TSND151; ATR-Promotions Inc)^a^ were attached to the anterior superior iliac spine, mid-thigh, and mid-lower leg on the exercising side. Pairs of disposable Ag/AgCl surface electrodes (SE-EXP-LEC60; Sekisui Kasei Co, Ltd)^b^ were placed with an interelectrode distance of 20 mm (center-to-center) on 8 muscles: ipsilateral RF, contralateral BF, bilateral RA, IO, and EO.^15^^,^^16^ The skin was cleaned with ethanol and skin treatment gel (Nuprep; Weaver and Company)^c^ before electrode placement. Measurements were conducted by trained staff who practiced the procedures on healthy individuals to reduce bias and ensure consistency.

**Fig 1:**
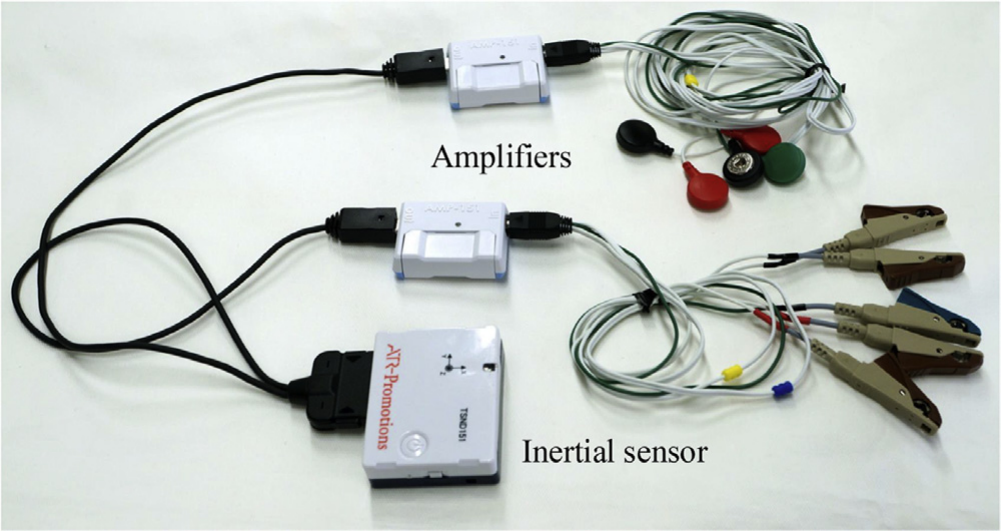
Measurement instruments used in this study.

**Fig 2:**
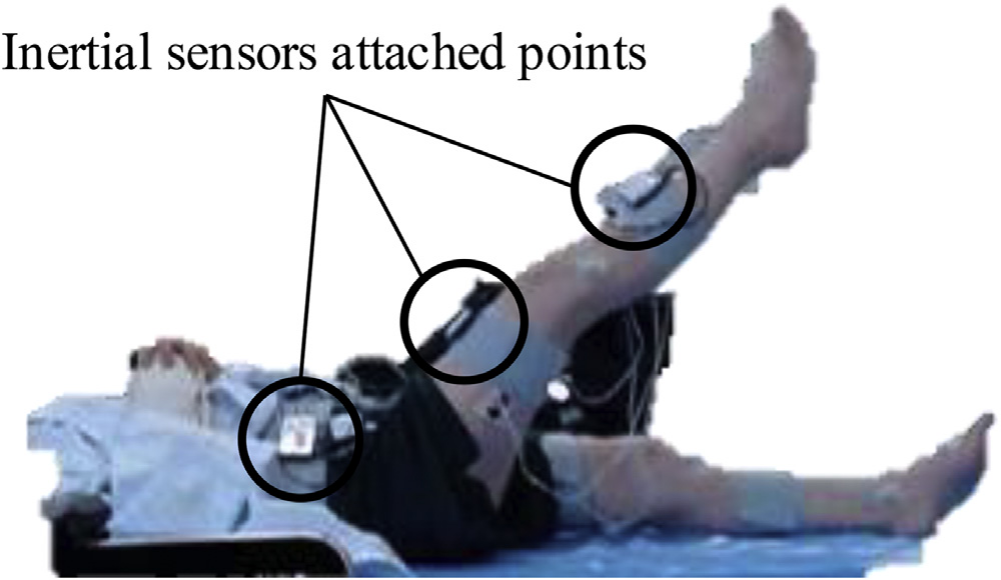
SLR measurement scene. Abbreviation: SLR, straight-leg raising.

Data were measured synchronously at a sampling rate of 1000 Hz using amplifiers to measure biological signals (AMP-151; ATR-Promotions Inc)^d^ and operated on software (ALTIMA; ATR-Promotions Inc)^e^ using a laptop. Raw EMG signals were processed using a bandpass filter with cutoff frequencies of 10 and 500 Hz and a common mode rejection ratio>90 dB and were amplified and collected. The instruments and measurement scenarios are shown in Fig 1, Fig 2.

### Data analysis

The SLR start point was identified based on the pitch angle from the lower leg. We analyzed the data from the start point to 10 s after the exercise. We applied a 1-Hz high-pass filter to the acceleration to remove the gravitational component using a zero-lag fourth-order Butterworth filter. We then calculated the combined acceleration for each site, and the mean values of the 3 trials were used for statistical analysis.

Each EMG was bandpass filtered between 5 and 450 Hz using a zero-lag fourth-order Butterworth filter. To normalize the amplitudes of EMG, each participant performed 3 trials of bilateral simultaneous SLR as a submaximal voluntary contraction for 5 s. We calculated the root mean square (RMS) of each muscle every 50 ms over the central 3 s. The RMS values obtained during the nondominant SLR were then normalized using the average RMS values across the 3 trials and expressed as a percentage. This is an index of muscle activity, with higher values indicating muscle activation closer to maximum effort. For the frequency analysis, we performed a continuous wavelet transform with 20 scales. We used the complex Morlet wavelet as the mother wavelet and set the bandwidth parameter and center frequency to 1 Hz.^17^ We computed the cumulative sum of the squared amplitude values at each time point and the median frequency (MDF) of each muscle. The MDF is an indicator of muscle fatigue and fiber recruitment patterns, with lower MDF values reflecting greater muscle fatigue and preferential recruitment of type I fibers, which are associated with lower force generation. The average of 3 trials was used for statistical analysis. The analyses were performed using Python 3.10.1 and its modules: NumPy 1.26.2, Pandas 1.3.5, PyWavelets 1.2.0, and SciPy 1.12.0.

### Statistical analysis

Twenty patients each in the WD and WI groups were analyzed. We used the Fisher exact test to compare sex and AIS grade ratios between the groups. Regarding other basic information, the unpaired *t* test or Mann–Whitney *U* test was applied after checking the normal distribution and homogeneity of variance of each data point for group comparison. These analyses were performed with a 2-tailed test using a modified R Commander 4.2.2.^18^

We followed 4 steps to construct a prediction model to evaluate the level of walking independence based on basic information and measured data. The objective variable was the binary classification into WD and WI groups according to scores for Mobility Indoors of the SCIM. First, 26 variables were selected based on the results of previous studies and the hypotheses of this study: sex, age, body mass index, total length of stay, number of measurement days since injury, UEMS, LEMS, combined accelerations at 3 sites, and RMS and MDF at 8 muscles. Because the sample size for each group was 20, we had to narrow these variables to no more than 2 for use in the prediction model. Therefore, we performed lasso-regularized logistic regression with 10-fold cross-validation to determine the optimal lambda value for regularization with the smallest error.^19^ We then conducted a second lasso-regularized logistic regression with the optimal lambda and extracted the variables for which the coefficients were not zero. Second, we calculated Pearson’s correlation coefficients among the variables extracted using the second lasso regression. Third, a logistic regression model with 10-fold cross-validation was constructed to predict walking independence by selecting any 2 variables that were not significantly correlated. As multiple prediction models were constructed using this procedure, their suitability for the data was compared using the Akaike information criterion (AIC). Finally, the lowest AIC model was used to evaluate the 95% confidence interval (CI) and odds ratio for each variable, predictive accuracy, and area under the receiver operating characteristic curve (AUC). These analyses were performed using R version 4.1.2. The level of statistical significance was set at *P*<.05. We summarized the descriptive statistics as means and standard deviations.

## Results

All patients completed SLR measurements. Four and 8 patients in the WD and WI groups, respectively, had some form of bone injury such as fractures of the vertebral body, transverse processes, and spinous processes. Except for 1 patient in the WD group, all patients underwent either emergency or scheduled surgery. The LEMS was missing in 1 patient from each group and UEMS in 1 patient from the WI group. Additionally, 1 patient from each group had missing RMS because of a lack of EMG during bilateral SLR. The remaining data were used for between-group comparisons. The basic information for each group is presented in table 1. After hospitalization and initial treatment, the patients were diagnosed with AIS grades C or D at the start of rehabilitation, except for 1 patient with grade A in the WD group. This patient was initially graded A but was included after subsequent recovery to grade C. At discharge or transfer, all patients in the WI group and 90% of those in the WD group had grade D, indicating comparable rates between the groups. The lengths of stay ranged from 11 to 90 days in the WI group and from 7 to 91 days in the WD group. Additionally, 3 patients in the WI group and 8 in the WD group stayed for more than 1 month. The UEMS and LEMS and the Mobility Indoors scores were all significantly higher in the WI group (all *P*<.01).

**Table 1:** Basic information for each group

	WD Group (n=20)	WI Group (n=20)	*P* Values
Age (y)	70.7±14.4	63.7±15.4	.134
Gender	18 M, 2 F	16 M, 4 F	.661
Height (cm)	164.1±7.9	163.1±8.2	.683
Weight (kg)	65.5±11.2	63.2±13.0	.558
AIS grade at start of rehabilitation	1 A, 7 C, 12 D	2 C, 18 D	.064
AIS grade at discharge or transfer	2 C, 18 D	20 D	.487
SLR acquisition since injury (d)	5.8±6.0	6.9±5.1	.316
Measurement since injury (d)	17.4±10.0	14.0±5.2	.272
Independent walking since injury (d)	None	12.0±7.9	None
Total length of stay (d)	31.2±20.9	25.7±16.5	.597
UEMS (from 0 to 50 points)*	27.2±8.7	37.6±9.8	**<.01**
LEMS (from 0 to 50 points)†	38.2±6.4	48.4±3.1	**<.01**
Score for Mobility Indoors of SCIM (from 0 to 8 points)	1.2±1.5	7.5±1.3	**<.01**

Abbreviations: AIS, American Spinal Cord Injury Association Impairment Scale; F, female; LEMS, lower extremity motor score; M, male; SCIM, Spinal Cord Independence Measure; UEMS, upper extremity motor score; WD, walking dependent, WI, walking independent.

Bold: *P* Value was less than 0.05.

⁎ Missing 1 patient in the WI group.

† Missing 1 patient in each group.

**Fig 3:**
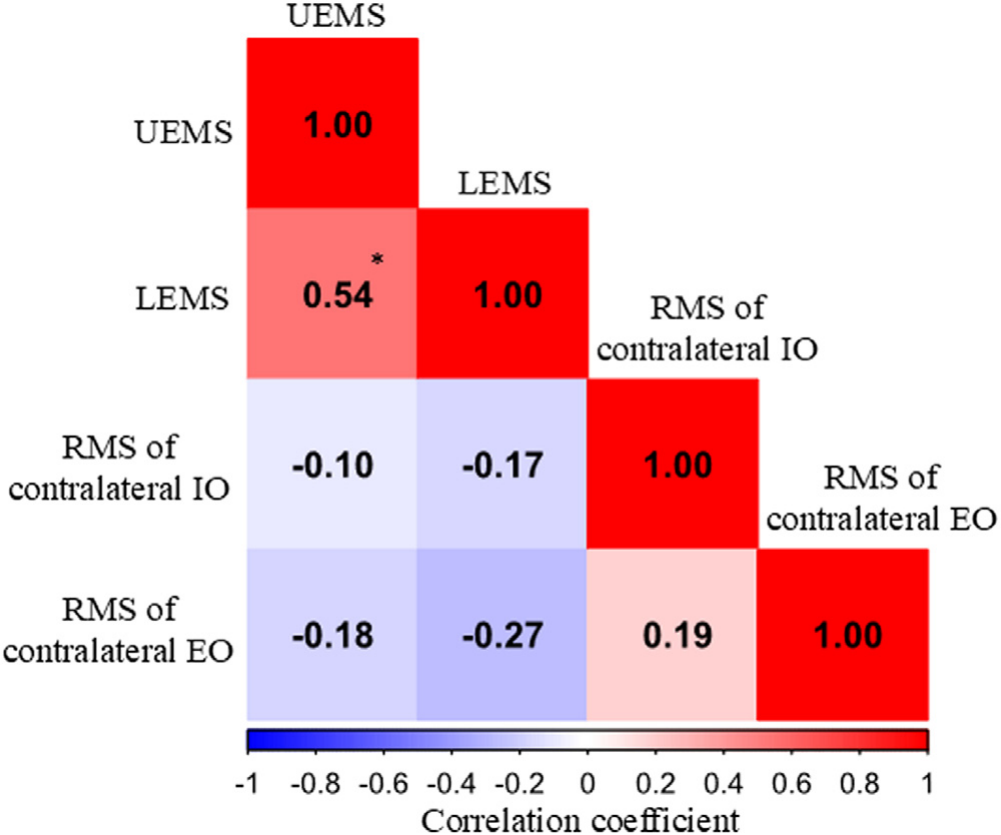
Heatmap of the correlation matrix among extracted variables. *P<.01. Abbreviations: EO, external oblique IO, internal oblique; LEMS, lower extremity motor score; RMS, root mean square; UEMS, upper extremity motor score.

**Fig 4:**
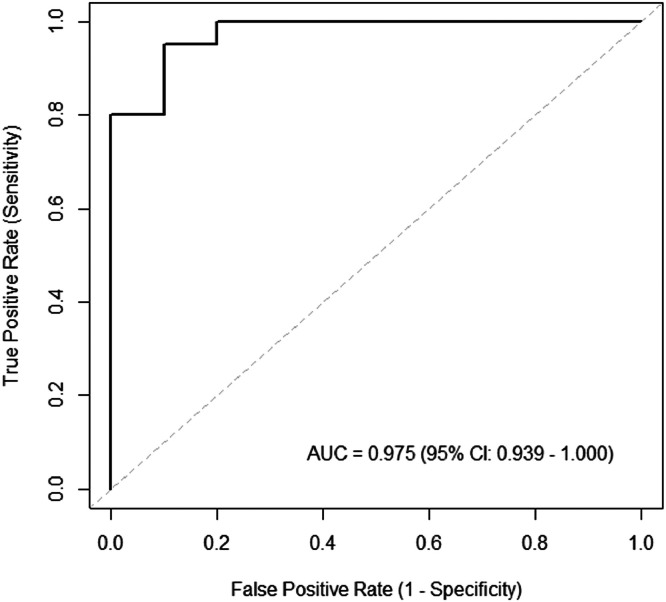
Receiver operating characteristic curve for the lowest AIC model. Abbreviations: AIC, Akaike information criterion; AUC, area under the receiver operating characteristic curve; CI, confidence interval.

The missing values were interpolated using the overall median to construct a prediction model. A total of 4 nonzero variables were extracted from the second lasso-regularized logistic regression: UEMS (standardized beta: −0.010), LEMS (standardized beta: −0.219), and RMS of the contralateral IO and EO (standardized beta: 0.109 and 2.380). A heatmap of the correlation matrix for these 4 variables is shown in figure 3. Significant correlations were only observed between the UEMS and LEMS (*r*=0.54, *P*<.01). Therefore, we selected 2 variables for logistic regression, 1 each from the UEMS and LEMS and from the RMS of the contralateral IO and EO, and the RMS of both contralateral IO and EO, resulting in a total of 5 prediction models. The results showed that the model with the LEMS and RMS of the contralateral EO had the lowest AIC of 22.24. Other AIC values were 47.67 for the UEMS and RMS of the contralateral IO, 40.27 for the UEMS and RMS of the contralateral EO, 31.65 for the LEMS and RMS of the contralateral IO, and 46.71 for the RMS of both contralateral IO and EO. The lowest AIC model parameters and confusion matrix are listed in Table 2, Table 3. Both the LEMS and RMS of the contralateral EO were significant predictors (*P*=.019 and .034). The predictive accuracy, sensitivity, and specificity calculated using the confusion matrix were 0.875, 0.850, and 0.900, respectively. The AUC was 0.975 (95% CI, 0.939-1.000; fig 4).

**Table 2:** Parameters of the lowest AIC model

	Estimate	Standard Error	Odds Ratio	95% CI for Odds Ratio	*P* Values
Intercept	0.530	0.695	1.478	0.466-8.512	.446
LEMS	−5.367	2.295	0.006	8.080^−6^-0.108	**.019**
RMS of contralateral EO	2.909	1.373	16.213	2.402-728.300	**.034**

Abbreviations: AIC, Akaike information criterion; CI: confidence interval; EO, external oblique; LEMS, lower extremity motor score; RMS, root mean square.

Bold: *P* Value was less than 0.05.

**Table 3:** Confusion matrix of the lowest AIC model

		Predicted Label	
		Positive (WD Group)	Negative (WI Group)
True label	Positive (WD group)	17	3
	Negative (WI group)	2	18

Abbreviations: AIC, Akaike information criterion; WD, walking dependent; WI, walking independent.

## Discussion

This study attempted to predict walking independence in patients with ICCI at discharge or transfer from an acute-care hospital using multivariate analysis. The results showed that the model with the LEMS and RMS of the contralateral EO best fitted the prediction of walking independence.

The model including the LEMS provided a better fit than the model including the UEMS for predicting walking independence. A previous study reported that LEMS evaluated in the subacute phase, between 16 and 40 days postinjury, was most strongly associated with walking ability at 6 months or later postinjury in both incomplete paraplegic and tetraplegic patients.^4^ Our results partially support this finding, as the LEMS was assessed during the acute to subacute phase, at a mean of 12.0±7.1 days postinjury, with assessment times ranging from 1 to 28 days. Whereas walking independence was evaluated only at discharge or transfer from the acute-care hospital, at a mean of 28.0±18.8 days postinjury, with evaluation times ranging from 7 to 91 days. Nevertheless, the LEMS is considered a particularly important factor directly related to walking ability, regardless of the timing of the walking assessment. By contrast, the UEMS, may be less important than lower extremity. This may be related to the recent increase in central cord syndrome.^20^ The results of this study, in which the UEMS was approximately 10 points lower than the LEMS in both groups, suggest that many patients may have had central cord syndrome, although not all patients were directly diagnosed. Thus, the degree of residual lower extremity muscle strength may be more important for walking acquisition than for impaired upper extremity muscle strength.

In another study, a LEMS of 41.5 points or higher was associated with independent community ambulation at a mean of 142 days postinjury, with times ranging from 7 to 277 days.^3^ In our study, the WD group had a LEMS of 38.2±6.4 points. Among the remaining 19 patients (excluding 1 with missing data) in the WD group, only 6 had an LEMS of 41.5 points or higher. By contrast, in the WI group, the LEMS was 48.4±3.1 points, and 18 of the 19 patients had a LEMS of 41.5 or higher, a noticeably greater number than in the WD group. Because the maximum LEMS score per side is 25 points, achieving a total of 41.5 or higher requires a high score, specifically, at least 4 out of 5 points per muscle on both sides. Additionally, although this study included patients who had acquired SLR only on the nondominant leg, all patients in the WI group could perform SLR on both sides, whereas only 5 patients in the WD group achieved SLR on the nondominant leg alone. Therefore, sufficient lower extremity muscle strength to perform SLR bilaterally may be a prerequisite for achieving walking independence, upon which the evaluation of muscle activity during SLR may become meaningful.

By contrast, the RMS of the contralateral EO was also significant factors associated with walking independence. Healthy individuals have been reported predominant activation of the ipsilateral RF, contralateral BF, and bilateral IO, with relatively less activation of the EO, which tends to be more active on the ipsilateral side during SLR.^5^^,^^7^ By contrast, patients with chronic pain exhibit increased bilateral EO activity.^9^ Thus, increased contralateral EO activity may reflect abnormal SLR muscle activation, which could be a compensatory response to lower extremity muscle weakness.

### Study limitations

This study had several limitations. First, the sample size was relatively small to conduct multivariate analysis. Additionally, because of missing data, we used imputed data for analysis. Second, the study was conducted at a single hospital, which may limit the generalizability of the findings to other populations or settings. Third, sensory scores were not included in analysis because 13 of 40 patients had missing values, making their inclusion impractical. Finally, although we predicted walking independence at the acute-care hospital, the long-term prognosis remains unknown.

## Conclusions

A higher LEMS and lower RMS of the contralateral EO during nondominant SLR are significant predictors of walking independence at discharge or transfer from an acute-care hospital in patients with ICCI.

## Suppliers

a. TSND151; ATR-Promotions Inc.
b. SE-EXP-LEC60; Sekisui Kasei Co, Ltd.
c. Nuprep; Weaver and Company.
d. AMP-151; ATR-Promotions Inc.
e. ALTIMA; ATR-Promotions Inc.
